# Verbal Learning and Memory Deficits across Neurological and Neuropsychiatric Disorders: Insights from an ENIGMA Mega Analysis

**DOI:** 10.3390/brainsci14070669

**Published:** 2024-06-29

**Authors:** Eamonn Kennedy, Spencer W. Liebel, Hannah M. Lindsey, Shashank Vadlamani, Pui-Wa Lei, Maheen M. Adamson, Martin Alda, Silvia Alonso-Lana, Tim J. Anderson, Celso Arango, Robert F. Asarnow, Mihai Avram, Rosa Ayesa-Arriola, Talin Babikian, Nerisa Banaj, Laura J. Bird, Stefan Borgwardt, Amy Brodtmann, Katharina Brosch, Karen Caeyenberghs, Vince D. Calhoun, Nancy D. Chiaravalloti, David X. Cifu, Benedicto Crespo-Facorro, John C. Dalrymple-Alford, Kristen Dams-O’Connor, Udo Dannlowski, David Darby, Nicholas Davenport, John DeLuca, Covadonga M. Diaz-Caneja, Seth G. Disner, Ekaterina Dobryakova, Stefan Ehrlich, Carrie Esopenko, Fabio Ferrarelli, Lea E. Frank, Carol E. Franz, Paola Fuentes-Claramonte, Helen Genova, Christopher C. Giza, Janik Goltermann, Dominik Grotegerd, Marius Gruber, Alfonso Gutierrez-Zotes, Minji Ha, Jan Haavik, Charles Hinkin, Kristen R. Hoskinson, Daniela Hubl, Andrei Irimia, Andreas Jansen, Michael Kaess, Xiaojian Kang, Kimbra Kenney, Barbora Keřková, Mohamed Salah Khlif, Minah Kim, Jochen Kindler, Tilo Kircher, Karolina Knížková, Knut K. Kolskår, Denise Krch, William S. Kremen, Taylor Kuhn, Veena Kumari, Junsoo Kwon, Roberto Langella, Sarah Laskowitz, Jungha Lee, Jean Lengenfelder, Victoria Liou-Johnson, Sara M. Lippa, Marianne Løvstad, Astri J. Lundervold, Cassandra Marotta, Craig A. Marquardt, Paulo Mattos, Ahmad Mayeli, Carrie R. McDonald, Susanne Meinert, Tracy R. Melzer, Jessica Merchán-Naranjo, Chantal Michel, Rajendra A. Morey, Benson Mwangi, Daniel J. Myall, Igor Nenadić, Mary R. Newsome, Abraham Nunes, Terence O’Brien, Viola Oertel, John Ollinger, Alexander Olsen, Victor Ortiz García de la Foz, Mustafa Ozmen, Heath Pardoe, Marise Parent, Fabrizio Piras, Federica Piras, Edith Pomarol-Clotet, Jonathan Repple, Geneviève Richard, Jonathan Rodriguez, Mabel Rodriguez, Kelly Rootes-Murdy, Jared Rowland, Nicholas P. Ryan, Raymond Salvador, Anne-Marthe Sanders, Andre Schmidt, Jair C. Soares, Gianfranco Spalleta, Filip Španiel, Scott R. Sponheim, Alena Stasenko, Frederike Stein, Benjamin Straube, April Thames, Florian Thomas-Odenthal, Sophia I. Thomopoulos, Erin B. Tone, Ivan Torres, Maya Troyanskaya, Jessica A. Turner, Kristine M. Ulrichsen, Guillermo Umpierrez, Daniela Vecchio, Elisabet Vilella, Lucy Vivash, William C. Walker, Emilio Werden, Lars T. Westlye, Krista Wild, Adrian Wroblewski, Mon-Ju Wu, Glenn R. Wylie, Lakshmi N. Yatham, Giovana B. Zunta-Soares, Paul M. Thompson, Mary Jo Pugh, David F. Tate, Frank G. Hillary, Elisabeth A. Wilde, Emily L. Dennis

**Affiliations:** 1 Department of Neurology, University of Utah School of Medicine, Salt Lake City, UT 84132, USA; eamonn.kennedy@utah.edu (E.K.); spencer.liebel@hsc.utah.edu (S.W.L.); hannah.lindsey@hsc.utah.edu (H.M.L.); shashank.vadlamani@utah.edu (S.V.); m.newsome@hsc.utah.edu (M.R.N.); maryjo.pugh@hsc.utah.edu (M.J.P.); david.tate@hsc.utah.edu (D.F.T.); elisabeth.wilde@hsc.utah.edu (E.A.W.); 2 Division of Epidemiology, University of Utah, Salt Lake City, UT 84108, USA; mustafa.ozmen@antalya.edu.tr; 3 George E Wahlen Veterans Affairs Medical Center, Salt Lake City, UT 84148, USA; 4 Department of Educational Psychology, Counseling, and Special Education, Pennsylvania State University, University Park, PA 16802, USA; puiwalei@gmail.com; 5 WRIISC-WOMEN & Rehabilitation Department, VA Palo Alto, Palo Alto, CA 94304, USAxiaojian.kang@va.gov (X.K.); vlioujohnson@stanford.edu (V.L.-J.); 6 Neurosurgery, Stanford School of Medicine, Stanford, CA 94305, USA; 7 Department of Psychiatry, Dalhousie University, Halifax, NS B3H 4R2, Canada; malda@dal.ca (M.A.); nunes@dal.ca (A.N.); 8 FIDMAG Research Foundation, 08025 Barcelona, Spain; sa.lana85@gmail.com (S.A.-L.); pfuentes@fidmag.org (P.F.-C.); epomarol-clotet@fidmag.org (E.P.-C.); rsalvador@fidmag.org (R.S.); 9 Centro Investigación Biomédica en Red Salud Mental (CIBERSAM), 28029 Madrid, Spain; carango@hggm.es (C.A.); rayesa@idival.org (R.A.-A.); bcrespo@us.es (B.C.-F.); gutierreza@peremata.com (A.G.-Z.); vilellae@peremata.com (E.V.); 10 Ace Alzheimer Center Barcelona, Universitat Internacional de Catalunya, 08022 Barcelona, Spain; 11 Department of Medicine, University of Otago, Christchurch 8011, New Zealand; tim.anderson@otago.ac.nz (T.J.A.); john.dalrymple-alford@canterbury.ac.nz (J.C.D.-A.); tracy.melzer@otago.ac.nz (T.R.M.); 12 New Zealand Brain Research Institute, Christchurch 8011, New Zealand; daniel.myall@nzbri.org; 13 Department of Neurology, Te Whatu Ora–Health New Zealand Waitaha Canterbury, Christchurch 8011, New Zealand; 14 Department of Child and Adolescent Psychiatry, Institute of Psychiatry and Mental Health, Hospital General Universitario Gregorio Marañón, Instituto de Investigación Sanitaria Gregorio Marañón (IiSGM), School of Medicine, Universidad Complutense, 28040 Madrid, Spain; covadonga.martinez@iisgm.com (C.M.D.-C.); jmerchan@iisgm.com (J.M.-N.); 15 Department of Psychiatry and Biobehavioral Sciences, Semel Institute for Neuroscience and Human Behavior, University of California Los Angeles, Los Angeles, CA 90095, USA; rasarnow@mednet.ucla.edu (R.F.A.); tbabikian@mednet.ucla.edu (T.B.); chinkin@ucla.edu (C.H.); tkuhn@mednet.ucla.edu (T.K.); athames@mednet.ucla.edu (A.T.); 16 Brain Research Institute, University of California Los Angeles, Los Angeles, CA 90095, USA; 17 Department of Psychology, University of California Los Angeles, Los Angeles, CA 90095, USA; 18 Translational Psychiatry, Department of Psychiatry and Psychotherapy, University of Lübeck, 23562 Lübeck, Germany; mihai.avram@uksh.de (M.A.); stefan.borgwardt@uksh.de (S.B.); 19 Department of Psychiatry, Marqués de Valdecilla University Hospital, Instituto de Investigación Sanitaria Valdecilla (IDIVAL), School of Medicine, University of Cantabria, 39008 Santander, Spain; newvtro@gmail.com; 20 UCLA Steve Tisch BrainSPORT Program, University of California Los Angeles, Los Angeles, CA 90095, USA; cgiza@mednet.ucla.edu; 21 Laboratory of Neuropsychiatry, Santa Lucia Foundation IRCCS, 00179 Rome, Italy; n.banaj@hsantalucia.it (N.B.); r.langella@hsantalucia.it (R.L.); f.piras@hsantalucia.it (F.P.); federica.piras@hsantalucia.it (F.P.); g.spalletta@hsantalucia.it (G.S.); d.vecchio@hsantalucia.it (D.V.); 22 School of Clinical Sciences, Monash University, Clayton, VIC 3800, Australia; laura.bird1@monash.edu; 23 Center of Brain, Behaviour and Metabolism (CBBM), University of Lübeck, 23562 Lübeck, Germany; 24 Cognitive Health Initiative, School of Translational Medicine, Monash University, Melbourne, VIC 3800, Australia; amy.brodtmann@monash.edu; 25 Department of Medicine, Royal Melbourne Hospital, Melbourne, VIC 3050, Australia; terence.obrien@monash.edu; 26 Department of Psychiatry and Psychotherapy, University of Marburg, 35032 Marburg, Germany; kbrosch@northwell.edu (K.B.); andreas.jansen@med.uni-marburg.de (A.J.); kircher2@staff.uni-marburg.de (T.K.); nenadic@staff.uni-marburg.de (I.N.); steinfre@staff.uni-marburg.de (F.S.); straubeb@staff.uni-marburg.de (B.S.); thomasod@staff.uni-marburg.de (F.T.-O.); adrian90wroblewski@gmail.com (A.W.); 27 Institute of Behavioral Science, Feinstein Institutes for Medical Research, Manhasset, NY 11030, USA; 28 Cognitive Neuroscience Unit, School of Psychology, Deakin University, Burwood, VIC 3125, Australia; k.caeyenberghs@deakin.edu.au; 29 Tri-Institutional Center for Translational Research in Neuroimaging and Data Science (TReNDS), Georgia State, Georgia Tech, Emory University, Atlanta, GA 30322, USA; vcalhoun@gsu.edu (V.D.C.); rootesmurdy@gmail.com (K.R.-M.); 30 Centers for Neuropsychology, Neuroscience & Traumatic Brain Injury Research, Kessler Foundation, East Hanover, NJ 07936, USA; nchiaravalloti@kesslerfoundation.org; 31 Department of Physical Medicine & Rehabilitation, Rutgers, New Jersey Medical School, Newark, NJ 07103, USA; jdeluca@kesslerfoundation.org (J.D.); edobryakova@kesslerfoundation.org (E.D.); hgenova@kesslerfoundation.org (H.G.); dkrch@kesslerfoundation.org (D.K.); jlengenfelder@kesslerfoundation.org (J.L.); gwylie@kesslerfoundation.org (G.R.W.); 32 Rehabilitation Medicine Department, National Institutes of Health Clinical Center, Bethesda, MD 20892, USA; david.cifu@vcuhealth.org; 33 Department of Psychiatry, Virgen del Rocio University Hospital, School of Medicine, University of Seville, IBIS, 41013 Seville, Spain; 34 School of Psychology, Speech and Hearing, University of Canterbury, Christchurch 8041, New Zealand; 35 Department of Rehabilitation and Human Performance, Icahn School of Medicine at Mount Sinai, New York, NY 10029, USAcarrie.esopenko@mountsinai.org (C.E.); 36 Department of Neurology, Icahn School of Medicine at Mount Sinai, New York, NY 10029, USA; 37 Institute for Translational Psychiatry, University of Münster, 48149 Münster, Germany; dannlow@uni-muenster.de (U.D.); jgolt@uni-muenster.de (J.G.); dominik.grotegerd@wwu.de (D.G.); marius.gruber@uni-muenster.de (M.G.); s.meinert@uni-muenster.de (S.M.); repple@uni-frankfurt.de (J.R.); 38 Department of Neuroscience, Monash University, Melbourne, VIC 3800, Australia; david.darby@monash.edu (D.D.); cassandra.marotta@monash.edu (C.M.); lucy.vivash@monash.edu (L.V.); 39 Department of Neurology, Alfred Health, Melbourne, VIC 3004, Australia; 40 The Florey Institute of Neuroscience and Mental Health, Melbourne, VIC 3052, Australia; heath.pardoe@florey.edu.au (H.P.); werdene@unimelb.edu.au (E.W.); 41 Department of Psychiatry and Behavioral Sciences, University of Minnesota Medical School, Minneapolis, MN 55455, USA; daven012@umn.edu (N.D.); disne014@umn.edu (S.G.D.); marqu310@umn.edu (C.A.M.); sponh001@umn.edu (S.R.S.); 42 Minneapolis VA Health Care System, Minneapolis, MN 55417, USA; 43 Kessler Foundation, East Hanover, NJ 07936, USA; 44 Center for Traumatic Brain Injury, Kessler Foundation, East Hanover, NJ 07936, USA; 45 Translational Developmental Neuroscience Section, Division of Psychological and Social Medicine and Developmental Neurosciences, Faculty of Medicine, Technische Universität Dresden, 01307 Dresden, Germany; stefan.ehrlich@uniklinikum-dresden.de; 46 Eating Disorders Research and Treatment Center, Department of Child and Adolescent Psychiatry, Faculty of Medicine, Technische Universität Dresden, 01307 Dresden, Germany; 47 Department of Psychiatry, University of Pittsburgh, Pittsburgh, PA 15213, USA; ferrarellif@upmc.edu (F.F.); mayelia@upmc.edu (A.M.); 48 Department of Psychology, University of Oregon, Eugene, OR 97403, USA; 49 Department of Psychiatry, University of California San Diego, La Jolla, CA 92093, USA; cfranz@ucsd.edu (C.E.F.); wkremen@ucsd.edu (W.S.K.); jorodrig@ucsd.edu (J.R.); astasenko@ucsd.edu (A.S.); 50 Center for Behavior Genetics of Aging, University of California San Diego, La Jolla, CA 92093, USA; 51 Center for Autism Research, Kessler Foundation, East Hanover, NJ 07936, USA; 52 Department of Pediatrics, Division of Neurology, UCLA Mattel Children’s Hospital, Los Angeles, CA 90095, USA; 53 Department of Neurosurgery, David Geffen School of Medicine at UCLA, Los Angeles, CA 90095, USA; 54 Department of Psychiatry, Psychosomatic Medicine and Psychotherapy, University Hospital Frankfurt, Goethe University, 60590 Frankfurt, Germany; 55 Hospital Universitari Institut Pere Mata, 43007 Tarragona, Spain; 56 Institut d’Investiació Sanitària Pere Virgili-CERCA, Universitat Rovira i Virgili, 43007 Tarragona, Spain; 57 Department of Brain and Cognitive Sciences, Seoul National University College of Natural Sciences, Seoul 08826, Republic of Korea; hamin746@snu.ac.kr (M.H.); kwonjs@snu.ac.kr (J.K.); jungha.lee@snu.ac.kr (J.L.); 58 Department of Biomedicine, University of Bergen, 5007 Bergen, Norway; jan.haavik@uib.no; 59 Division of Psychiatry, Haukeland University Hospital, 5021 Bergen, Norway; 60 Center for Biobehavioral Health, The Abigail Wexner Research Institute at Nationwide Children’s Hospital, Columbus, OH 43205, USA; kristen.hoskinson@nationwidechildrens.org; 61 Section of Pediatrics, The Ohio State University College of Medicine, Columbus, OH 43210, USA; 62 Translational Research Centre, University Hospital of Psychiatry and Psychotherapy, University of Bern, 3000 Bern, Switzerland; daniela.hubl@unibe.ch; 63 Ethel Percy Andrus Gerontology Center, Leonard Davis School of Gerontology, University of Southern California, Los Angeles, CA 90089, USA; irimia@usc.edu; 64 Department of Biomedical Engineering, Viterbi School of Engineering, University of Southern California, Los Angeles, CA 90089, USA; 65 Department of Quantitative & Computational Biology, Dornsife College of Arts & Sciences, University of Southern California, Los Angeles, CA 90089, USA; 66 University Hospital of Child and Adolescent Psychiatry and Psychotherapy, University of Bern, 3000 Bern, Switzerland; michael.kaess@upd.ch (M.K.); jochen.kindler@unibe.ch (J.K.); chantal.michel@upd.unibe.ch (C.M.); 67 Clinic of Child and Adolescent Psychiatry, Centre of Psychosocial Medicine, University of Heidelberg, 69120 Heidelberg, Germany; 68 Department of Neurology, Uniformed Services University of the Health Sciences, Bethesda, MD 20814, USA; kimbra.kenney@usuhs.edu; 69 National Institute of Mental Health, 250 67 Klecany, Czech Republic; barbora.kerkova@nudz.cz (B.K.); karolina.knizkova@nudz.cz (K.K.); mabel.rodriguez@nudz.cz (M.R.); filip.spaniel@nudz.cz (F.Š.); 70 Cognitive Health Initiative, Central Clinical School, Monash University, Melbourne, VIC 3800, Australia; mohamed.khlif@monash.edu; 71 Department of Neuropsychiatry, Seoul National University Hospital, Seoul 03080, Republic of Korea; verte82@snu.ac.kr; 72 Department of Psychiatry, Seoul National University College of Medicine, Seoul 03080, Republic of Korea; 73 Department of Psychiatry, First Faculty of Medicine, Charles University and General University Hospital, 128 00 Prague, Czech Republic; 74 Norwegian Centre for Mental Disorders Research (NORMENT), Division of Mental Health and Addiction, Oslo University Hospital, 0424 Oslo, Norway; kolskaar@gmail.com (K.K.K.); genevieve.richard@medisin.uio.no (G.R.); anne-marthe.sanders@sunnaas.no (A.-M.S.); kristine.moe.ulrichsen@sunnaas.no (K.M.U.); l.t.westlye@psykologi.uio.no (L.T.W.); 75 Department of Psychology, University of Oslo, 0373 Oslo, Norway; mar.lovstad@gmail.com; 76 Department of Research, Sunnaas Rehabilitation Hospital, 1450 Nesodden, Norway; 77 Department of Life Sciences, College of Health, Medicine and Life Sciences, Brunel University London, Uxbridge UB8 3PH, UK; veena.kumari@kcl.ac.uk; 78 Brain Imaging and Analysis Center, Duke University, Durham, NC 27710, USA; laskowitz.sarah@gmail.com (S.L.); rajendra.morey@duke.edu (R.A.M.); 79 National Intrepid Center of Excellence, Walter Reed National Military Medical Center, Bethesda, MD 20814, USA; sara.m.lippa.civ@health.mil (S.M.L.); john.m.ollinger.civ@mail.mil (J.O.); 80 Department of Neuroscience, Uniformed Services University of the Health Sciences, Bethesda, MD 20814, USA; 81 Department of Biological and Medical Psychology, University of Bergen, 5007 Bergen, Norway; astri.lundervold@uib.no; 82 Institute D’Or for Research and Education (IDOR), São Paulo 04501-000, Brazil; paulomattosmd@gmail.com; 83 Department of Radiation Medicine and Applied Sciences and Psychiatry, University of California San Diego, La Jolla, CA 92093, USA; camcdonald@health.ucsd.edu; 84 Center for Multimodal Imaging and Genetics, University of California San Diego, La Jolla, CA 92093, USA; 85 Institute for Translational Neuroscience, University of Münster, 48149 Münster, Germany; 86 VISN 6 MIRECC, Durham VA, Durham, NC 27705, USA; 87 Center of Excellence on Mood Disorders, Louis A Faillace, MD Department of Psychiatry and Behavioral Sciences, McGovern Medical School, The University of Texas Health Science Center at Houston, Houston, TX 77030, USA; benson.irungu@uth.tmc.edu (B.M.); jair.c.soares@uth.tmc.edu (J.C.S.); mon-ju.wu@uth.tmc.edu (M.-J.W.); giovana.b.zuntasoares@uth.tmc.edu (G.B.Z.-S.); 88 Faculty of Computer Science, Dalhousie University, Halifax, NS B3H 4R2, Canada; 89 Department of Neuroscience, The School of Translational Medicine, Alfred Health, Monash University, Melbourne VIC 3004, Australia; 90 Department of Psychiatry, Psychosomatic Medicine and Psychotherapy, Frankfurt University, 60590 Frankfurt, Germany; viola.oertel@unimedizin-ffm.de; 91 Department of Psychology, Norwegian University of Science and Technology, 7491 Trondheim, Norway; alexander.olsen@ntnu.no; 92 Department of Physical Medicine and Rehabilitation, St Olavs Hospital, Trondheim University Hospital, 7006 Trondheim, Norway; 93 NorHEAD—Norwegian Centre for Headache Research, 7491 Trondheim, Norway; 94 Department of Electrical and Electronics Engineering, Antalya Bilim University, 07190 Antalya, Turkey; 95 Neuroscience Institute & Department of Psychology, Georgia State University, Atlanta, GA 30303, USA; mbparent@gsu.edu; 96 WG (Bill) Hefner VA Medical Center, Salisbury, NC 28144, USA; jared.rowland@va.gov; 97 Department of Neurobiology & Anatomy, Wake Forest School of Medicine, Winston-Salem, NC 27157, USA; 98 VA Mid-Atlantic Mental Illness Research Education and Clinical Center (MA-MIRECC), Durham, NC 27705, USA; 99 Cognitive Neuroscience Unit, School of Psychology, Deakin University, Geelong, VIC 3220, Australia; nicholas.ryan@deakin.edu.au; 100 Department of Paediatrics, The University of Melbourne, Parkville, VIC 3052, Australia; 101 Department of Psychiatry (UPK), University of Basel, 4002 Basel, Switzerland; andre.schmidt@unibas.ch; 102 3rd Faculty of Medicine, Charles University, 100 00 Prague, Czech Republic; 103 Imaging Genetics Center, Stevens Neuroimaging & Informatics Institute, Keck School of Medicine of the University of Southern California, Marina del Rey, CA 90292, USA; sthomopo@usc.edu (S.I.T.); thompson@ini.usc.edu (P.M.T.); 104 Department of Psychology, Georgia State University, Atlanta, GA 30303, USA; etone@gsu.edu; 105 Department of Psychiatry, University of British Columbia, Vancouver, BC V6T 1Z4, Canada; ivan.torres@ubc.ca (I.T.); l.yatham@ubc.ca (L.N.Y.); 106 British Columbia Mental Health and Substance Use Services Research Institute, Vancouver, BC V5Z 1M9, Canada; 107 Michael E DeBakey Veterans Affairs Medical Center, Houston, TX 77030, USA; mayat@bcm.edu; 108 H Ben Taub Department of Physical Medicine and Rehabilitation, Baylor College of Medicine, Houston, TX 77030, USA; 109 Psychiatry and Behavioral Health, Ohio State Wexner Medical Center, Columbus, OH 43210, USA; jessica.turner@osumc.edu; 110 Division of Endocrinology, Emory University School of Medicine, Atlanta, GA 30322, USA; geumpie@emory.edu; 111 Department of Physical Medicine and Rehabilitation, Virginia Commonwealth University, Richmond, VA 23298, USA; william.walker@vcuhealth.org; 112 Richmond Veterans Affairs (VA) Medical Center, Central Virginia VA Health Care System, Richmond, VA 23249, USA; 113 KG Jebsen Center for Neurodevelopmental Disorders, University of Oslo, 0372 Oslo, Norway; 114 Department of Psychology, Phoenix VA Health Care System, Phoenix, AZ 85012, USA; krista.wild@va.gov; 115 Rocco Ortenzio Neuroimaging Center, Kessler Foundation, East Hanover, NJ 07936, USA; 116 Departments of Neurology, Pediatrics, Psychiatry, Radiology, Engineering, and Ophthalmology, University of Southern California, Los Angeles, CA 90089, USA; 117 Department of Psychology, Penn State University, State College, PA 16801, USA; fhillary@psu.edu; 118 Department of Neurology, Hershey Medical Center, State College, PA 16801, USA; 119 Social Life and Engineering Science Imaging Center, Penn State University, State College, PA 16801, USA

**Keywords:** verbal learning, memory, dementia, depression, Parkinson’s disease, schizophrenia, stroke, traumatic brain injury, bipolar disorder, attention-deficit/hyperactivity disorder

## Abstract

Deficits in memory performance have been linked to a wide range of neurological and neuropsychiatric conditions. While many studies have assessed the memory impacts of individual conditions, this study considers a broader perspective by evaluating how memory recall is differentially associated with nine common neuropsychiatric conditions using data drawn from 55 international studies, aggregating 15,883 unique participants aged 15–90. The effects of dementia, mild cognitive impairment, Parkinson’s disease, traumatic brain injury, stroke, depression, attention-deficit/hyperactivity disorder (ADHD), schizophrenia, and bipolar disorder on immediate, short-, and long-delay verbal learning and memory (VLM) scores were estimated relative to matched healthy individuals. Random forest models identified age, years of education, and site as important VLM covariates. A Bayesian harmonization approach was used to isolate and remove site effects. Regression estimated the adjusted association of each clinical group with VLM scores. Memory deficits were strongly associated with dementia and schizophrenia (*p* < 0.001), while neither depression nor ADHD showed consistent associations with VLM scores (*p* > 0.05). Differences associated with clinical conditions were larger for longer delayed recall duration items. By comparing VLM across clinical conditions, this study provides a foundation for enhanced diagnostic precision and offers new insights into disease management of comorbid disorders.

## 1. Introduction

Memory performance is a core cognitive function and a key determinant of overall health and quality of life [1,2]. Numerous neurological, neurodevelopmental, and neuropsychiatric disorders have been linked to memory deficits, including, but not limited to, dementia [3], mild cognitive impairment (MCI) [4], Parkinson’s disease (PD) [5], traumatic brain injury (TBI) [6], stroke [7], depression [8], attention-deficit/hyperactivity disorder (ADHD) [9], schizophrenia [10], and bipolar disorder (BD) [11]. However, comprehensive evaluations of cognitive measures have typically been limited to meta-analyses of distinct clinical groups. Little work has directly assessed the relative impact of various neurological and neuropsychiatric disorders on verbal learning and memory (VLM) within one unified framework, in part due to the complexities of examining heterogeneous clinical populations with distinct research priorities and treatment gaps [12,13]. Understanding the differential impact(s) of clinical conditions on VLM could yield tangible benefits, including enhanced diagnostic precision and new insights into disease management for individuals with comorbid neurological and neuropsychiatric disorders [14,15].

### 1.1. Neurological Disorders and VLM

This study aggregated assessments of VLM based on single-word list learning tasks. VLM assessments involve immediate, short-term, and long-term memory items, which reflect different temporal stages or types of memory processing related to the encoding, storage, and retrieval of verbal information [16]. These stages are governed by neurological mechanisms with varying susceptibility to clinical conditions.

This study focused on VLM specifically because they are directly relevant to many everyday activities, such as conversation and instruction, and they are also sensitive measures of the cognitive impact of many diseases. Unlike disease-specific measures, VLM tools are also versatile and can be used with validity across a range of distinct clinical conditions. For example, Alzheimer’s disease and related dementias (AD/RD) have profound and extensively studied effects on various aspects of VLM [17], particularly impaired encoding abilities [18]. Amnestic MCI, which can be a prodromal phase of AD/RD [19], also exerts well-documented effects on VLM [20]. Yet, even within these well-characterized diseases, complex and heterogeneous memory performance is often observed across individuals and disease subtypes [21,22], and some individuals with AD have relatively preserved memory in the early disease stages [23]. Dementia subtypes, such as vascular dementia (VaD) and frontotemporal dementia (FTD) can demonstrate variation in VLM across affected individuals [24]. Some individuals with FTD may have difficulties with the storage, consolidation, and later recall of verbal information [25,26], whereas VaD associations with VLM can depend on the extent and location of vascular pathology [27]. PD and related neurodegenerative disorders often present with memory difficulties in the later stages of disease progression [28], while some studies have found VLM changes in newly diagnosed PD patients [29]. 

TBI along the severity spectrum has been linked to cognitive changes, including VLM deficits [30,31]. Research also suggests an increased risk for dementia among TBI survivors [32]. A wide range of conditions can emerge and persist after TBI, although the cognitive effects of TBI depend on injury severity and recency [33,34]. VLM impairments also occur in other acquired brain injury populations, such as stroke, in which various aspects of memory are affected depending on the location and severity of injury [35].

### 1.2. Neuropsychiatric Disorders and VLM

Despite intensive research, the precise impacts of various common neuropsychiatric disorders on cognitive functioning remain unclear [36]. For example, prior work found that memory deficits were not consistently associated with depression [37], while other studies found that major depressive disorder (MDD) and depression-related symptoms negatively affect memory [38]. These findings are further complicated by the comorbid relationship between depression and other conditions that may have confounding effects on memory performance, such as TBI [39]. Impairments in working memory are a core feature of ADHD, and individuals with ADHD may experience reduced performance on long-term memory tasks, while short-term memory deficits, when detected, tend to be less pronounced [40,41]. 

Schizophrenia is associated with decreased cognitive performance, and patients with schizophrenia consistently show substantial deficits in VLM, which has been supported by imaging studies [42,43,44,45]. Schizophrenia has also been associated with disproportionate episodic memory impairments [46]. Similarly, impaired relational memory (recall of concept and object associations) is considered a core deficit in schizophrenia [47] and has been linked to the early stages of psychosis [48]. BD has also been associated with VLM impairments [49], particularly in late-stage BD [50], which is likely due to its relation to the number of previous manic episodes and psychiatric hospitalizations [51].

The heterogeneous pathologies, disease courses, and consequences of these neurological and neuropsychiatric disorders strongly suggest a differential effect among patient populations on VLM scores. To date, comprehensive evaluations of cognitive measures have typically been limited to meta-analyses of single clinical groups. This study considered a broader perspective by quantifying item-level VLM disruptions across a range of common neurological and neuropsychiatric disorders in a large sample using a standardized analysis. This was achieved by taking advantage of new methods for the harmonization of distinct VLM assessments [52], as well as methods to isolate and remove the influence of site on VLM scores. We hypothesized that neurological and neuropsychiatric disorders would display differential relationships with VLM performance. Score declines per group were further broken out into age- and sex/gender-stratified models. By aggregating a large sample of item-level assessments drawn from diverse settings, this study reveals both absolute and comparative insights across neurological and neuropsychiatric disorders.

## 2. Materials and Methods

### 2.1. Data Source

**Figure 1:**
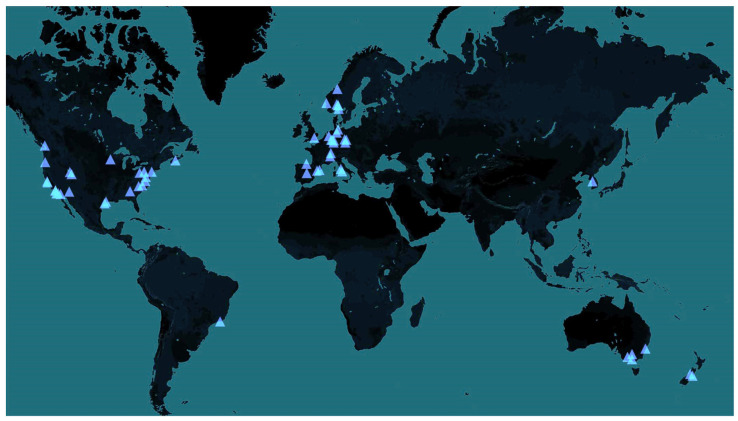
World map showing the locations of participating collaborator institutions (triangles). Details for each sub-study are provided in Supplementary Table S1.

Following prior work [53], we aggregated secondary de-identified data, drawing from 55 studies of adults contributed by collaborators in the Psychiatric Genomics Consortium (PGC), the Enhancing NeuroImaging Genetics through Meta-Analysis Consortium (ENIGMA) working groups (including the ENIGMA Brain Injury working group), and the Long-term Impact of Military-relevant Brain Injury Consortium–Chronic Effects of Neurotrauma Consortium (LIMBIC-CENC). A world map of the locations of participating institutions is shown in Figure 1.

### 2.2. Clinical Disorders

The inclusion of clinical disorders was based on available samples with clear diagnostic classifications. Comprehensive details for the exclusion and inclusion criteria for each group per site are detailed in Supplementary Table S1, including pharmacological considerations. AD/RD, MCI, PD, TBI, stroke, MDD, ADHD, schizophrenia/psychosis, and BD were included. Each clinical condition group was aggregated by including participants who had the same primary diagnoses across studies. For example, the ‘Schizophrenia/Psychosis’ group was defined to include any participants who had a primary diagnosis listed as one of the following eight terms: *Schizophrenia*, *Psychosis*, *Clinical High Risk for Psychosis*, *Early Course Schizophrenia*, *First Episode Psychosis*, *SCZ Patient*, *SZ*, *Schizoaffective*, and *Ultra High Risk of Psychosis*. Similarly, TBI included individuals with primary diagnosis terms that included *Concussion*, *mild TBI*, *moderate/severe TBI*, and *penetrating TBI.* Controls were defined as those with a primary classification listed as one of the following: *Healthy control*, *Control*, *Unexposed*, *Negative*, *HN controls*, and *HC*. This process excluded 727 individuals with ambiguous diagnoses.

### 2.3. Measures

The California Verbal Learning Test, 2nd edition (CVLT-II) [54] is a validated tool for assessing VLM performance. The CVLT-II uses a 16-word list drawn from 4 categories that is presented verbally over 5 consecutive learning trials. The CVLT-II includes an assessment of verbal learning using immediate (no delay), short- (following a distractor task), and long- (20 min) delay free verbal recall trials and a distractor list. Free recall scores range from 0 to 16 on each of the five immediate recall/learning trials, as well as the short- and long-delay trials. A total score across the five learning trials (Trials 1–5) ranges from 0 to 80 words recalled. Raw scores from the first immediate recall trial (T1), Trials 1–5, short-delay free recall (SDFR), and long-delay free recall (LDFR) were chosen as the four primary outcomes of interest. While some studies administered measures at multiple visits, only data collected during initial assessments were included.

Prior to analysis, we performed a harmonization of scores across the CVLT-II, Rey Auditory Verbal Learning Task (RAVLT) [55], and Hopkins Verbal Learning Task–Revised (HVLT-R) [56,57] using a previously validated score conversion table tool (available at https://verbal-learning.halfpipe.group, accessed on 25 June 2024) [53]. This process ensured assessments were aligned to CVLT-II item scales; therefore, all measures were harmonized prior to analysis. To briefly describe the two underlying measures, the HVLT-R is a validated and relatively short measure of VLM deficits. The HVLT-R uses 12-word lists, which are drawn from three semantic categories, and it uses a small (*N* = 24) total pool of words for scoring. HVLT-R does not use a distractor list for immediate recall, and it does not assess short-delay recall performance. The HVLT-R has three consecutive learning trials. The RAVLT is a validated measure of VLM deficit. The RAVLT draws from random, semantically unrelated words and employs a 15-word list length and a distractor list, along with short- and 30-min delay recall trials.

### 2.4. Data Processing

Datasets from all sources were cleaned, standardized, and aggregated. Nearest neighbor imputation was used to infer missing data, and overall missingness was low (< 5%). In a study involving many data sources, individual source-specific effects can reduce statistical power and lead to inaccurate results. Therefore, following prior work, an empirical Bayes method was used to isolate and remove source-specific effects from scores while preserving covariate effects [53]. Using harmonization, we can assure uniformity above that of any single study by aggregating and comparing many datasets. This process allowed us to identify and remove biases inherent in each single data source, e.g., due to the influence of specific inclusion criteria on scores. Only controls were used to estimate site effects.

### 2.5. Design

Participants were assigned to clinical groups by primary clinical diagnosis. Imbalance across clinical groups was mitigated using a two-step design that included both matching and covariate adjustment [58]. Using a nearest neighbor algorithm, each clinical group was matched 1:1 with healthy controls using covariates of age, sex/gender, years of education, and geographic region. This was required because the total control group was not representative of all clinical groups. For example, controls were on average younger than participants in the AD/RD cohort. After matching, linear regression was used to estimate group differences in T1, Trials 1–5, SDFR, and LDFR scores, adjusting for covariates. 

### 2.6. Covariates

Covariates included age, sex/gender, years of education, geographical region, language of assessment, and site/study source. The raw demographic information available varied across sites. For example, some studies recorded sex while others recorded gender; these data were aggregated into a single sex/gender variable. The data included eight languages (Czech, English, German, Italian, Korean, Norwegian, Portuguese, and Spanish) that were tested for relative impact on scores. Prior research in this sample found no significant racial differences associated with VLM scores [53], so we elected to not include race variables in our analysis. 

### 2.7. Statistical Analysis

All analyses were conducted in Python 3. Geographical region, language of assessment, and site are highly interrelated factors. Therefore, to preliminarily screen for important covariates that may require subsequent adjustment, random forest models were used due to their robustness against collinearity [59]. The *sklearn* python package was used to estimate variable importances with random forest models. Linear regression was used to test for significance across groups after adjusting for the covariates identified with high feature importances in random forest models. Percentage differences in words recalled between clinical groups and matched controls were calculated by converting adjusted coefficients to percentages of words recalled. In age- and sex/gender-stratified models, confidence bounds for significance were estimated by random sampling and averaging effects, comparing within control groups.

## 3. Results

The total dataset consisted of 15,883 participants drawn from 55 sites; 36.8% were female, and the median age was 42 years. At the time of assessment, the cohort had received an average of 13.7 ± 3.0 years of education. As shown in Table 1, the clinical groups with the largest sample sizes were TBI (*n* = 4867), schizophrenia (*n* = 1962), and MDD (*n* = 1063), while the MCI (*n* = 233) and ADHD (*n* = 126) groups had the smallest sample sizes. Controls (*n* = 5713) were drawn from 43 out of 55 studies. Table 1 shows clear trends across groups, but additional adjustments were required. The AD/RD, MCI, and PD clinical groups tended to be older and had a higher percentage of females. The TBI and SZ groups were younger and included more males. The TBI, BD, and ADHD groups were the most educated clinical groups, on average. 

**Figure 2:**
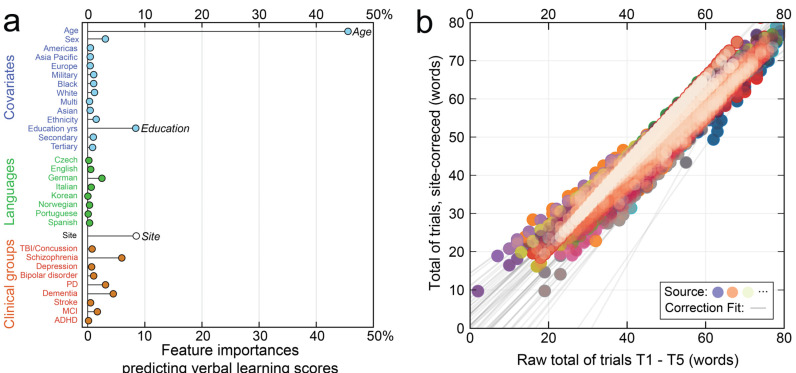
Preliminary screening of covariates by random forest models. In (**a**), feature importances identified age, education, and site as the most important variables for adjustment. In (**b**), raw Trials 1–5 scores are plotted against Trials 1–5 scores after source correction (color indicates origin study). Gray lines show the fits used to correct for each site.

Determining the influence of language, study, and covariates on scores is challenging due to collinearity. To address this issue, Figure 2a shows a stem plot of random forest model feature importances predicting VLM scores averaged across items. The model achieved 80.7% explained variance, with age (45.6%), education (8.5%), and origin study (8.4%) estimated as the three most important features. By contrast, the eight language variables (Czech, English, German, Italian, Korean, Norwegian, Portuguese, and Spanish) were minimally predictive of scores. This analysis suggested that source correction followed by regression on covariates was sufficient to accurately estimate performance associations with clinical conditions. Correction for site effects was performed using ComBat-GAM [60], an empirical Bayes method that was specified to tolerate nonlinearities in score responses as a function of age. The fits used to correct for each site are shown as gray lines in Figure 2b. The harmonization process was performed for each item.

After site correction, linear regression was used to estimate covariates (Table 2). The model comparators used were: age, 16–35; sex/gender, male; geographic region, Americas; and education, primary level. Age-related differences in scores were significant across all VLM items (*p* < 0.001). Compared to those aged 16–35 years, the number of words recalled on both SDFR and LDFR declined by more than 20% for those aged 65+. Age-related differences were also significant for the immediate recall items, T1, and Trials 1–5 (*p* < 0.001). Women showed higher VLM performance across all measures compared to men, ranging from +2.9% to +7.6% (*p* < 0.001 for all items). Geographic region showed inconsistent associations and mixed significance across items. Education level was positively associated with VLM performance across all items (*p* < 0.001). 

**Figure 3:**
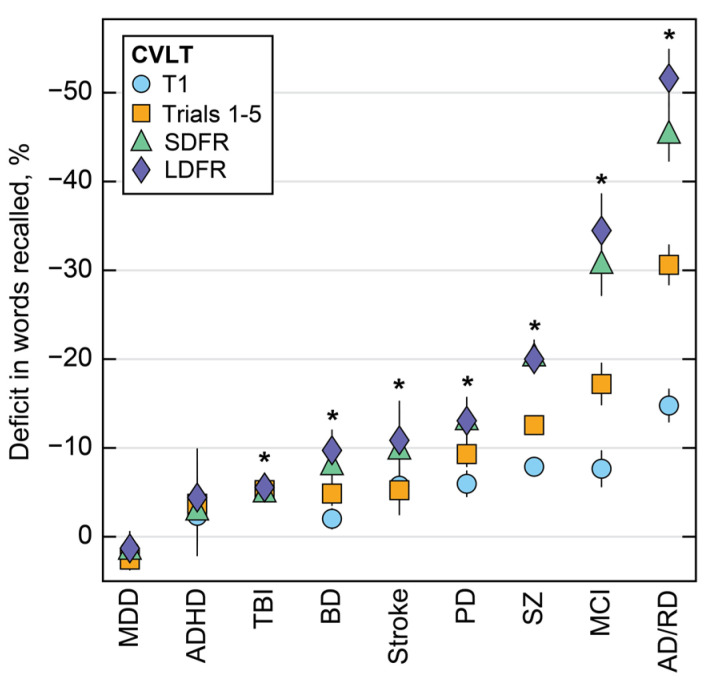
Percentage changes in words recalled are shown for each clinical group relative to matched controls. Numerical data for this figure are available in Supplementary Table S2. MDD = major depressive disorder. ADHD = attention-deficit/hyperactivity disorder. TBI = traumatic brain injury. BD = bipolar disorder. PD = Parkinson’s disease. SZ = schizophrenia. MCI = mild cognitive impairment. AD/RD = Alzheimer’s disease and related dementias. T1 = immediate recall trial 1. Trials 1–5 = total score across immediate recall/learning trials 1–5. SDFR = short-delay free recall. LDFR = long-delay free recall. * significant at *p* < 0.01 for all four items.

Percentage differences in words recalled between clinical groups and matched controls were estimated from regression coefficients (Figure 3). The disorder with the largest score difference relative to controls was AD/RD (T1: −14.8%, SDFR: −45.6%, LDFR: −51.6%), while ADHD and MDD showed the smallest effects overall on the percentage of words recalled. All clinical groups were significantly different from controls across all VLM items (*p* < 0.001), except for ADHD and MDD, which did not exhibit consistently different recall from matched controls (*p* > 0.05). The strength of association varied across items and delay durations. In Figure 3, a consistent pattern emerged where declines in LDFR (purple diamonds) exceeded T1 deficits (blue circles). This association was most pronounced for AD/RD, which exhibited a 37% difference between the percentage of words recalled on T1 and LDFR items compared to controls.

**Figure 4:**
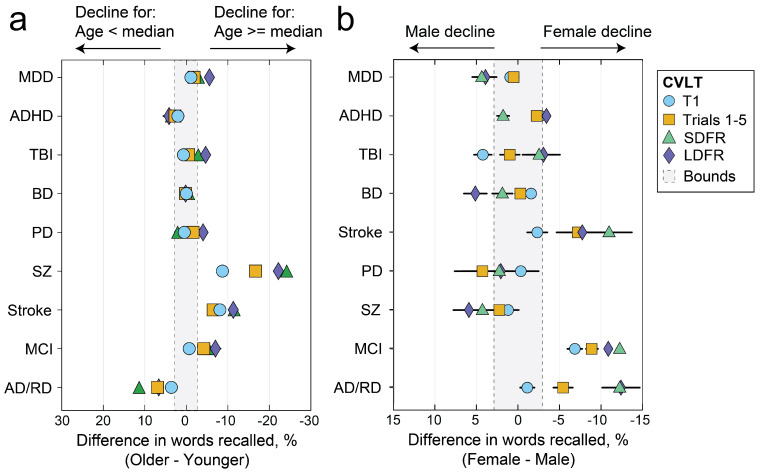
Age- (**a**) and sex/gender- (**b**) stratified percentage score differences across female/male and above/below median age groups relative to matched controls. MDD = major depressive disorder. ADHD = attention-deficit/hyperactivity disorder. TBI = traumatic brain injury. BD = bipolar disorder. PD = Parkinson’s disease. SZ = schizophrenia. MCI = mild cognitive impairment. AD/RD = Alzheimer’s disease and related dementias. T1 = immediate recall trial 1. Trials 1–5 = total score across immediate recall/learning trials 1–5. SDFR = short-delay free recall. LDFR = long-delay free recall.

We additionally tested for sex/gender- and age-related differences in percentage change in VLM scores for each clinical group relative to matched controls. In Figure 4, declines for each clinical group relative to controls are shown with additional age and sex/gender stratification across older/younger and female/male groups. Median age per group was used as the stratification cutoff. Relative to matched controls, older patients with schizophrenia show greater declines than younger patients with schizophrenia. Females with stroke, MCI, and AD/RD showed worse declines than males with the same diagnoses. 

## 4. Discussion

Neurological and neuropsychiatric disorders have heterogeneous impacts on cognitive functioning [12,13]. Even in clinical diseases with well-established mechanisms of memory disruption, appreciable variations have been observed across studies and populations [8,10,17,18,21,26,36,40,49]. Understanding the differential impacts of clinical conditions on VLM performance may have tangible benefits, including new insights into disease management for individuals with comorbid neurological concerns with compounding memory effects. Drawing from a large repository of 15,883 unique participant assessments aggregated across 55 studies from multiple international consortia, this study investigated the impact of nine common neurological and neuropsychiatric disorders on VLM. We used existing harmonization tools to align scores drawn from different geographical regions and VLM assessments within a single unified analysis.

Our findings suggest that some, but not all, conditions were associated with abnormalities in VLM, confirming our a priori hypothesis. A pattern emerged in which the impact of clinical conditions on scores tended to increase with recall delay duration. This association was most pronounced for AD/RD, which exhibited a 37% difference in words recalled between the immediate and long-delay assessments. These data recapitulate that LDFR is an effective differentiator of disorders, particularly for medial temporal lobe pathology and hippocampal-dependent disorders. Our findings revealed that AD/RD, MCI, and schizophrenia were most strongly associated with VLM differences relative to controls, even after adjusting for covariates, including age. Although AD/RD and MCI are broad diagnostic categories, both have profound and heterogeneous effects on memory. Complex and heterogeneous memory performance is often observed across individuals and disease subtypes, such as amnestic MCI and non-amnestic MCI, amongst others [21]. 

Participants with TBI of any severity scored 5.1–5.5% lower on the VLM compared to controls, and while AD/RD showed large differences across items of different delay duration, TBI was associated with a narrower range of deficits across items. In the current analyses, all TBI severities were grouped together, and findings could vary across injury severity. 

Relative to controls, individuals with ADHD showed lower VLM scores on all items, but these differences were not consistently significant. Similarly, MDD effects were small (1–2%) compared to the associations of AD/RD and MCI (30–50%), and MDD was not consistently significantly associated with VLM. These mixed findings are consistent with the mixed literature on the impacts of MDD and ADHD on cognitive functioning [41]. 

Age and education have well-established associations with memory performance, and this study identified both as important covariates of VLM. Random forest models also identified origin site of assessment as the third most important determinant of VLM scores. Site effects cannot be observed or mitigated in single-site studies, but in this study, data were aggregated from a large number of studies so that harmonization models could isolate and remove individual site effects. In a secondary analysis stratifying clinical group associations with VLM by age and sex/gender, older patients with schizophrenia showed greater declines than younger patients with schizophrenia. Females with stroke, MCI, and AD/RD showed worse declines than males with the same diagnoses. 

The strengths of this study include a large sample size of more than 15,000 participants drawn from 55 diverse international data sources of VLM assessment. Random forest models that are robust against collinearity were used to identify key covariates requiring further adjustment. A large and diverse sample of over 5000 healthy control participants facilitated a good comparison pool for each clinical group. 

This study also had several limitations. While 13 countries and 8 languages were represented in our dataset, our data were skewed toward English-speaking samples from the Western hemisphere. Our ability to tease out the influence of disorders on VLM scores may also be confounded by unmeasured considerations, such as substance use disorder and other diseases that are highly comorbid in patients with neurocognitive disorders. We elected to frame the study using primary diagnosis groups and a matched design, but common comorbid concerns for TBI and depression, such as post-traumatic stress disorder, may contribute to the association between clinical conditions and VLM deficits. The effects of comorbidities on VLM is a standing limitation that would require deeper medical histories not consistently documented in each underlying study. Future work could explore whether there are meaningful interaction effects observed for patients with overlapping clinical conditions. While recognition test items are an important part of VLM testing, the harmonization of recognition memory indices was not implemented in existing harmonization models [53], and therefore, recognition memory items were not considered. Harmonization models, although previously validated, might introduce some bias for sources with low sample sizes. The data were aggregated from multiple sources with different instruments, settings, inclusion criteria, and assessment procedures [53]. However, a detailed understanding of study effects was not necessary to remove these effects in aggregate using Bayesian harmonization procedures. Data were largely drawn from studies of mild TBI, and the magnitude of associations with VLM may not reflect findings for more severe forms of TBI.

## 5. Conclusions

This study highlights the benefit of aggregating large sample sizes to determine how VLM deficits vary across the spectrum of neurological and neuropsychiatric disorders. Our findings suggest that some, but not all, conditions were associated with abnormalities in VLM. A pattern emerged suggesting that the impact of clinical conditions on scores tended to increase with recall delay duration. VLM performance was not consistently associated with a diagnosis of depression or ADHD on all items relative to matched controls, but it was significantly associated with all other conditions assessed. In a secondary analysis stratifying clinical group associations with VLM by age and sex/gender, older patients with schizophrenia showed greater declines than younger patients with schizophrenia. Females with stroke, MCI, and AD/RD showed worse declines than males with the same diagnoses. Beyond cognitive assessment, these findings may provide a means to identify novel diagnostic and mechanistic neuroimaging biomarkers of cognitive performance from large-scale, global open neuroscience initiatives with greater sensitivity and statistical power.

## Figures and Tables

**Table 1:** Characteristics and average CVLT-II scores for the nine clinical groups and controls. Average VLM performance of each group (i.e., mean score) is shown as raw scores and as percentage differences with respect to (% w.r.t.) the control group.

Clinical Group	Control	AD/RD	MCI	PD	TBI	Stroke	MDD	ADHD	SZ	BD
**N**	5713	276	233	628	4867	273	1063	126	1962	742
**# Studies**	43	5	4	3	23	3	7	3	19	12
**Age, %**										
16–35	43.5	0.0	0.9	0.0	25.7	3.3	49.8	56.3	64.6	40.5
35–65	42.1	9.1	12.9	36.3	67.0	31.1	49.2	42.9	34.9	56.5
65+	14.4	90.1	86.2	63.7	7.3	65.6	1.0	0.8	0.5	3.0
**Sex/Gender, %**										
Male	49.0	38.8	49.8	32.6	90.3	71.1	48.0	48.4	61.7	42.7
Female	51.0	61.2	50.2	67.4	9.7	28.9	62.0	51.6	38.3	57.3
**Region, %**										
Americas	54.8	79.7	70.8	0.0	100.0	0.0	3.2	40.5	17.9	39.2
Europe	41.5	18.1	21.5	37.7	0.0	51.3	96.8	58.7	79.6	60.8
Asia	3.7	2.2	7.7	62.3	0.0	58.7	0.0	0.8	2.5	0.0
**Education, %**										
Primary	10.0	50.2	42.5	51.6	0.7	30.6	7.6	15.9	25.4	21.3
Secondary	25.3	34.0	37.8	18.1	27.0	26.4	46.1	22.2	25.4	27.8
Tertiary	64.7	15.6	19.7	30.3	72.3	43.0	46.3	61.9	42.0	50.9
**Mean score**										
T1	6.5	3.0	4.1	4.2	5.8	4.6	7.2	6.4	5.4	6.2
*% w.r.t. controls*	*0%*	*−54%*	*−37%*	*−35%*	*−11%*	*−29%*	*11%*	*−2%*	*−17%*	*−5%*
Trials 1–5	52.4	21.6	31.7	29.4	47.7	41.3	55.8	53.2	43.8	48.9
*% w.r.t. controls*	*0%*	*−59%*	*−40%*	*−44%*	*−9%*	*−21%*	*6%*	*−2%*	*−16%*	*−7%*
SDFR	11.5	1.2	3.9	6.7	9.9	8.5	12.2	11.8	8.9	10.2
*% w.r.t. controls*	*0%*	*−90%*	*−66%*	*−42%*	*−14%*	*−26%*	*6%*	*−3%*	*−12%*	*−11%*
LDFR	11.8	1.2	3.8	6.4	10.2	8.9	12.8	12.2	9.1	10.7
*% w.r.t. controls*	*0%*	*−90%*	*−68%*	*−46%*	*−14%*	*−25%*	*8%*	*3%*	*−23%*	*−9%*

AD/RD = Alzheimer’s disease and related dementias. MCI = mild cognitive impairment. PD = Parkinson’s disease. TBI = traumatic brain injury. MDD = major depressive disorder. ADHD = attention-deficit/hyperactivity disorder. SZ = schizophrenia/psychosis. BD = bipolar disorder. T1 = first immediate recall trial. SDFR = short-delay free recall. LDFR = long-delay free recall.

**Table 2:** Blocked linear regressions estimating scores on immediate, short-, and long-delay verbal learning items for the whole cohort after site correction. Coefficients are shown as percentage associations with each item score.

Regression	T1	Trials 1–5	SDFR	LDFR
**N**	15,883	15,883	15,883	15,883
**Adj. R^2^**	19.2%	37.7%	32.9%	34.0%
**Age, % (CI)**				
16–35	–	–	–	–
35–65	−3.5 (−3.9, −3.1) ^†^	−6.0 (−6.5, −5.5) ^†^	−9.2 (−10.0, −8.5) ^†^	−9.7 (−10.5, −8.9) ^†^
65+	−8.2 (−8.9, −7.6) ^†^	−13.6 (−14.4, −12.8) ^†^	−21.8 (−23.1, −20.5) ^†^	−22.2 (−23.5, −20.9) ^†^
**Sex/Gender, % (CI)**				
Male	–	–	–	–
Female	2.9 (2.5, 3.3) ^†^	4.9 (4.4, 5.3) ^†^	7.0 (6.2, 7.8) ^†^	7.6 (6.9, 8.4) ^†^
**Region, % (CI)**				
Americas	–	–	–	–
Europe	−0.6 (−1.7, 0.5)	−7.0 (−8.3, −5.8) ^†^	−9.1 (−11.2, −7.0) ^†^	−8.5 (−10.6, −6.4) ^†^
Asia	1.3 (0.7, 1.8) ^†^	1.6 (1.0, 2.1) ^†^	0.7 (−0.3, 1.6)	1.4 (0.4, 2.4)
**Education, % (CI)**				
Primary	–	–	–	–
Secondary	3.2 (2.6, 3.8) ^†^	4.7 (3.9, 5.4) ^†^	6.2 (5.0, 7.5) ^†^	5.8 (4.6, 7.0) ^†^
Tertiary	4.5 (3.9, 5.1) ^†^	7.1 (6.4, 7.8) ^†^	10.5 (9.3, 11.6) ^†^	9.3 (8.1, 10.4) ^†^

T1 = immediate recall trial 1. Trials 1–5 = total score across immediate recall/learning trials 1–5. SDFR = short-delay free recall. LDFR = long-delay free recall. CI = confidence interval. ^†^ indicates significance at *p* < 0.001

## Data Availability

The raw data supporting the conclusions of this article and code used for analysis will be made available by the authors on reasonable request pending appropriate study approvals and data transfer agreements between participating institutions.

## Supplementary Materials

The following supporting information can be downloaded at: https://www.mdpi.com/article/10.3390/brainsci14070669/s1, Table S1: Inclusion/exclusion criteria for each data source; Table S2: Deficit in words recalled for each clinical condition relative to matched controls. Refs. [61,62,63,64,65,66,67,68,69,70,71,72,73,74,75,76,77,78,79,80,81,82,83,84,85,86,87,88,89,90,91,92,93,94,95,96,97,98,99,100] are cited in the Supplementary Materials.
